# Fidelity of SNP Array Genotyping Using Epstein Barr Virus-Transformed B-Lymphocyte Cell Lines: Implications for Genome-Wide Association Studies

**DOI:** 10.1371/journal.pone.0006915

**Published:** 2009-09-04

**Authors:** Joshua T. Herbeck, Geoffrey S. Gottlieb, Kim Wong, Roger Detels, John P. Phair, Charles R. Rinaldo, Lisa P. Jacobson, Joseph B. Margolick, James I. Mullins

**Affiliations:** 1 University of Washington School of Medicine, Seattle, Washington, United States of America; 2 University of California School of Public Health, Los Angeles, California, United States of America; 3 Northwestern University Medical School, Chicago, Illinois, United States of America; 4 University of Pittsburgh Graduate School of Public Health, Pittsburgh, Pennsylvania, United States of America; 5 Johns Hopkins University, Bloomberg School of Public Health, Baltimore, Maryland, United States of America; University of Montreal, Canada

## Abstract

**Background:**

As availability of primary cells can be limited for genetic studies of human disease, lymphoblastoid cell lines (LCL) are common sources of genomic DNA. LCL are created in a transformation process that entails *in vitro* infection of human B-lymphocytes with the Epstein-Barr Virus (EBV).

**Methodology/Principal Findings:**

To test for genotypic errors potentially induced by the Epstein-Barr Virus transformation process, we compared single nucleotide polymorphism (SNP) genotype calls in peripheral blood mononuclear cells (PBMC) and LCL from the same individuals. The average mismatch rate across 19 comparisons was 0.12% for SNPs with a population call rate of at least 95%, and 0.03% at SNPs with a call rate of at least 99%. Mismatch rates were not correlated across genotype subarrays run on all sample pairs.

**Conclusions/Significance:**

Genotypic discrepancies found in PBMC and LCL pairs were not significantly different than control pairs, and were not correlated across subarrays. These results suggest that mismatch rates are minimal with stringent quality control, and that most genotypic discrepancies are due to technical artifacts rather than the EBV transformation process. Thus, LCL likely constitute a reliable DNA source for host genotype analysis.

## Introduction

Advances in microarray technology have allowed high-throughput rapid genotyping of hundreds of thousands of single nucleotide polymorphisms (SNPs) across the human genome. These large sets of individual genotypes can be used for genome-wide association studies (GWAS), in which SNP allelic variation across a study population is tested for statistical associations with a particular disease phenotype. This method for studying the genetics of human disease has become widespread, and the genomic DNA necessary is generally provided by archived primary cells or tissue samples collected in prospective or longitudinal cohorts. As these samples are used for a wide range of studies and will become limited as more studies related to human disease are performed, the establishment of cell lines as permanent resources of genomic DNA is considered a potential solution. This process entails *in vitro* infection of human B-lymphocytes with the Epstein-Barr Virus, resulting in “immortalized” cell lines termed lymphoblastoid cell lines (LCL).

The feasibility of LCL for use in genetic studies has been evaluated primarily with regard to genomic copy number variation. Redon *et al*. compared copy number variations found in 268 HapMap (www.hapmap.org) LCL to copy number variations seen in each individual's blood cell-derived DNA [1]. They estimated that putative LCL-specific genomic errors accounted for less than 0.5% of observed deletions and considered LCL to be robust sources of genomic DNA for studies of copy number variation. Subsequent studies have supported the conclusion that LCL are likely to have little or minor effects on genomic structural variation (*e.g.* [2], [3], [4]).

With regard to gene expression and LCL, Choy *et al*. examined the utility of using HapMap LCL to identify expression quantitative trait loci (eQTL) that contribute to drug response phenotypes [5]. They found that non-genetic factors, such as the genomic EBV copy number, *in vitro* growth rate, and cellular ATP levels of individual LCL were more strongly associated with drug response and mRNA expression level phenotypes than any genotypic variation (genetic factors). The substantial noise from non-genetic factors impaired the ability to detect significant associations between genotypic variation and drug response or mRNA expression phenotypes. They noted that the non-genetic factors may be due to the EBV transformation process. In addition, Gimelbrant *et al*. and Plagnol *et al*. have documented that LCL exhibit extensive random, monoallelic expression [6], [7]. Plagnol *et al*. (2008) have suggested that LCL mRNA expression data may not be suitable for eQTL association analyses, given that LCL expression data can represent the random sampling of LCL clones with monoallelic expression not representative of the (source) cellular population as a whole.

LCL are commonly used in SNP analyses and genome-wide association studies, although the relationship between LCL genomic structural variation and genotype (SNP) fidelity is unclear. Redon *et al*. (2006) suggested that genomic duplications can result in Mendelian inconsistencies, and that genomic deletions can result in departures from Hardy-Weinberg equilibrium due to a lack of heterozygous genotypes. In this study, we attempted to quantify errors in SNP fidelity (genotypic discrepancies) that are potentially induced by the EBV transformation processes. To do so we compared the fidelity of SNP genotype calls in DNA obtained from paired samples of peripheral blood mononuclear cells (PBMC) and LCL from the same donor.

## Materials and Methods

### Samples

We compared the fidelity of SNP genotype calls in DNA obtained from paired samples of PBMC and LCL from 16 individuals of European American ancestry (mean age = 32.1 years) from the Multicenter AIDS Cohort Study (MACS). The MACS is an ongoing prospective study of the natural and treated histories of HIV-1 infection in homosexual and bisexual men conducted by study sites located in Baltimore, Chicago, Pittsburgh, and Los Angeles [8]. A total of 6,973 HIV-1 infected and uninfected men have been enrolled since 1984. The MACS collects plasma and PBMC, among other laboratory specimens and clinical data, from study participants at six-month intervals. In order to meet expected research demands on specimen availability, LCL were created from the PBMC from many participants.

The procedure for establishing LCL was adapted by the MACS from several previously described methods [9], [10], [11]. Briefly, B lymphocytes were separated from PBMC and incubated with Epstein-Barr Virus until immortalized. Immortalization was confirmed by continual increase in cell number, observation of cell blastogenesis and morphology, and detection of EBV antigens expressed in immortalized cell lines. After immortalization, transformed cells were grown in culture until they reached a concentration of 1×10^7^ to 1×10^8^ cells/ml. This stock culture served as the source for cell pellets of 5×10^6^ cells/vial stored at −80°C until use. These cells were not passaged after immortalization.

The participants in this analysis were selected from an ongoing study of associations between host genetic factors and prognostic phenotypes of HIV-1 infection. This study includes 210 MACS individuals, of which 118 were genotyped from PBMC samples and 71 were genotyped from LCL samples (due to limited PBMC availability). The selection of the individuals for the LCL genotypic fidelity study described here was random, and is not expected to affect the estimates of LCL fidelity. We genotyped both PBMC and LCL source DNA from 16 individuals. Four individuals were also genotyped in duplicate from the same DNA source (two from PBMC, two from LCL); these served as control analyses of genotypic fidelity, as well as adding three replicates of PBMC vs. LCL tests, bringing the total number of PBMC versus LCL mismatch comparisons to 19.

### Genotyping

DNA was extracted from PBMC and LCL cell pellets using the Qiagen QiaAmp blood mini-kit (Qiagen, Valencia, CA). We genotyped PBMC and LCL samples using the Affymetrix GeneChip Human Mapping 500 k array set (Affymetrix, Santa Clara, CA), which includes a combined 500,568 SNPs on two arrays, the 250 k Nsp I restriction enzyme assay (∼262,000 SNPs) and the 250 k Sty I restriction enzyme assay (∼238,000 SNPs). All samples were genotyped according to the Affymetrix protocol. In this approach, 250 ng of genomic DNA is first digested with a restriction enzyme (either Nsp I or Sty I), ligated to an adaptor, and amplified by PCR. The resulting amplicons are fragmented, labeled with biotinylated dideoxy ATP using terminal deoxynucleotidyl transferase, and hybridized to the array. Hybridization is detected by incubation with streptavidin-phycoerythrin conjugates, followed by scanning the array for phycoerythrin fluorescence and quantitation.

### Genotype Fidelity

SNP genotypes were called using the Affymetrix BRLMM algorithm [12], and the mean SNP array call rate for the combined 500 k array across all individuals was 98.5%. We define genotype fidelity as the SNP genotype concordances in LCL and PBMC from the same individual. We analyzed genotype fidelity using the genetic association software PLINK [13], with which we calculated identity-by-state pairwise distances for the 500 k combined array set and for each 250 k array separately. Prior to analysis, we subjected SNPs to multiple levels of quality control based on individual SNP call rates across the larger population data set of 210 individuals (including both the LCL and PBMC genotypes from the 16 individuals described here). Four sets of comparisons were done: one with no SNPs filtered; and three in which SNPs were filtered if they were not called (had no call or had an ambiguous genotype call) in at least 90%, 95%, and 99% or more of the population data set, respectively. Estimates of genomic copy number variation were inconsistent for this data set, as batch effects from different array processing dates were confounding.

It has been suggested [1] that genomic deletions will result in an increased number of SNPs that depart from Hardy-Weinberg equilibrium (HWE) due to a lack of heterozygous genotypes. While we could not directly test for genomic deletions in our study, we compared the number of SNPs deviating from HWE in LCL to the number deviating from HWE in PBMC. We tabulated SNPs that deviated from HWE at three levels of statistical significance, *P*<0.05, 0.01, and 0.001, in PBMC and LCL populations independently (in the larger population of 210 individuals, with no replicate samples included, after filtering by population call rate).

Care should be taken to distinguish the *population call rate* from the *array call rate*: the population call rate is the frequency of successful genotype calls for a given SNP across a sampled population; the array call rate is the frequency of successful genotype calls for a particular SNP array.

## Results

### Genotypic fidelity and SNP population call rate

We estimated genotypic fidelity of LCL genomic DNA by comparing SNP genotypes inferred from LCL and from the original PBMC, from the same donor, for 16 individuals. We additionally genotyped three individuals in replicate (for 19 total LCL and PBMC comparisons), and genotyped four individuals in duplicate from identical source DNA (two LCL, two PBMC).

**Figure 1:**
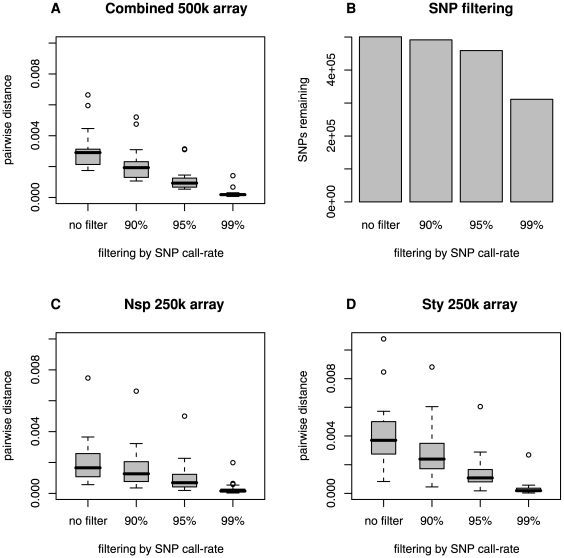
Genotypic fidelity of LCL. Genotypic fidelity is shown as mean pairwise distances among 19 paired comparisons of LCL and PBMC genotypes, for increasingly stringent SNP filtering by population call rate. A) Genotypic fidelity between LCL and PBMC source DNA from the same individual, estimated using the Affymetrix GeneChip Human Mapping 500 k Array set. B) SNP numbers remaining after filtering, shown for the combined 500 k array. C) Genotypic fidelity between LCL and PBMC source DNA from the same individual shown for the Nsp 250 k array. D) Genotypic fidelity between LCL and PBMC for the Sty 250 k array.

Genotypic fidelity increased with more stringent SNP calling quality control (Table 1, Figure 1). With SNP filtering at a 95% population call rate across the larger population study of 210 individuals, mean pairwise distance between PBMC and LCL genotypes was 0.12% for the 500 k combined array. This genotypic mismatch rate falls within the 0.10% to 0.30% mismatch rate reported by Affymetrix for replicate genotyping assays of the same purified DNA sample (after equivalent quality control). Further, mismatch rates at the 99% population call rate were ∼0.03%, equivalent to less than 100 mismatched SNPs in more than 300,000 genotype calls (Table 1). More stringent levels of quality control (increasing from no filtering upward to 99% population call rate) yielded significantly improved genotypic fidelity between PBMC and LCL genotypes (*P* = 3.82×10^−6^, Wilcoxon signed rank test, for the comparison of mismatch rates for no filtering and rates for filtering at the 99% population call rate level).

**Table 1:** Genotype fidelity between paired DNA samples, with SNPs filtered at increasing population call rates.

		filtering level:	no filtering	≥90%	≥95%	≥99%
		# SNPs remaining:	*500,568*	*491,525*	*458,913*	*311,241*
Comparison						
LCL vs PBMC		pairwise distance:				
	sample ID					
	594		0.00282	0.00193	0.00088	0.00013
	2046		0.00225	0.00151	0.00066	0.0001
	2048		0.00206	0.00132	0.00062	0.00012
	4195		0.00292	0.00106	0.00126	0.00026
	4195^a^		0.00221	0.00161	0.001	0.00019
	1881		0.00446	0.00309	0.00145	0.00026
	2061		0.0029	0.00193	0.00091	0.00018
	1854		0.00664	0.0052	0.00314	0.00067
	27		0.00196	0.00122	0.00054	0.00013
	2032		0.00175	0.00126	0.00061	0.00007
	2032^b^		0.00311	0.0021	0.00093	0.0001
	2035		0.00313	0.00215	0.0011	0.0003
	1988		0.00176	0.00128	0.00068	0.0001
	1988^c^		0.00332	0.00244	0.00124	0.00024
	1879		0.00595	0.00475	0.0031	0.00141
	2173		0.00299	0.00235	0.00133	0.00024
	976		0.00312	0.00228	0.00113	0.00022
	2037		0.00238	0.00161	0.00071	0.00012
	1880		0.00192	0.00126	0.00059	0.00013
		mean:	0.00303	0.00212	0.00115	0.00026
		s.d.:	0.00134	0.00114	0.00075	0.00031
Control duplicates						
	sample ID					
PBMC duplicate	4195		0.00168	0.00106	0.00053	0.00009
PBMC duplicate	2055		0.00352	0.00263	0.0014	0.00033
LCL duplicate	2032		0.00234	0.00157	0.0007	0.00008
LCL duplicate	1988		0.00305	0.00228	0.00121	0.00026
		mean:	0.00265	0.00189	0.00096	0.00019
		s.d.:	0.00081	0.0007	0.00041	0.00012

a LCL vs replicate PBMC.

b PBMC vs replicate LCL.

c PBMC vs replicate LCL.

**Figure 2:**
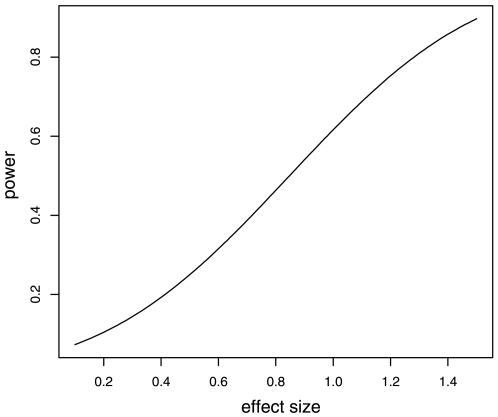
Power curve for comparisons of mismatch rates between LCL and PBMC pairs and duplicate pairs. Relationship between effect size, in units of (pooled) standard deviation, and statistical power, for a Mann-Whitney U test (one-sided, unequal sample sizes) with group samples sizes of 19 and 4. There is power of 0.62 to detect a difference in means of one standard deviation between LCL and PBMC pairs and duplicate pairs, equal to ∼90 SNPs out of >300,000 compared.

Comparisons of mismatch rates between LCL and PBMC pairs and duplicate pairs (duplicate genotyping of identical source DNA) revealed equivalent mean pairwise distances (*P* = 0.42, at the 99% population call rate level; Mann-Whitney U test, one sided, unequal sample sizes; Table 2). This test has a power of 0.62 to detect a difference of one standard deviation at a significance level of *P = *0.05 (Figure 2). Considering LCL and PBMC samples from the same individual genotyped with 500 k array and filtered for 99% population call rate, one standard deviation is equivalent to ∼90 mismatched SNPs out of >300,000 compared.

**Table 2:** Comparisons of mean SNP mismatch rates seen in LCL and PBMC pairs to rates seen in control (sample duplicate) pairs, at different levels of filtering by population call rate.

	Mismatch rates
	Duplicate pairs	LCL vs PBMC		LCL vs PBMC	
	(4 pairs)	(19 pair group)	*P* *	(16 pair group)	*P* *
no filtering	0.00265	0.00303	0.42	0.00302	0.48
≥90%	0.00189	0.00212	0.47	0.00217	0.45
≥95%	0.00096	0.00115	0.42	0.00115	0.48
≥99%	0.00019	0.00026	0.42	0.00027	0.41

* *P*-values for Mann-Whitney U test (one sided, unequal sample sizes) comparing pairwise distances seen in PMBC and LCL pairs from the same donor to pairwise distances seen in control pairs.

### Genotypic fidelity comparing Nsp and Sty 250 k arrays

The Affymetrix 500 k genotype array is composed of two separate arrays, each containing ∼250 k SNPs. In our study, all individuals were genotyped with both arrays. Overall, the Sty 250 k array had lower genotype call rates and lower genotypic fidelity than the Nsp 250 k array (Figure 1), although at 95% and 99% population call rate levels the genotypic fidelity approached equal levels.

In order to best investigate the association of genotypic errors with LCL, it is heuristic to compare the LCL and PBMC mismatch rates seen for Nsp and Sty arrays across multiple individuals. In effect, our experimental design included duplicate tests for every individual LCL and PBMC comparison, as each pair was genotyped with two arrays (Nsp and Sty). If mismatch rates seen with Nsp and Sty arrays are correlated, we can infer that genotypic discrepancies between LCL and PBMC pairs reflect underlying genomic errors potentially associated with LCL (because identical source DNA was genotyped on different arrays). If mismatch rates between Nsp and Sty arrays are unrelated, we can infer that observed genotypic discrepancies reflect technical artifacts related to the genotyping process. We found no correlation between LCL and PBMC mismatch rates seen for Nsp and Sty arrays (*P* = 0.919, Spearman's rank correlation coefficient, rho = −0.33).

### Genotypic fidelity and genotype array SNP call rate

**Figure 3:**
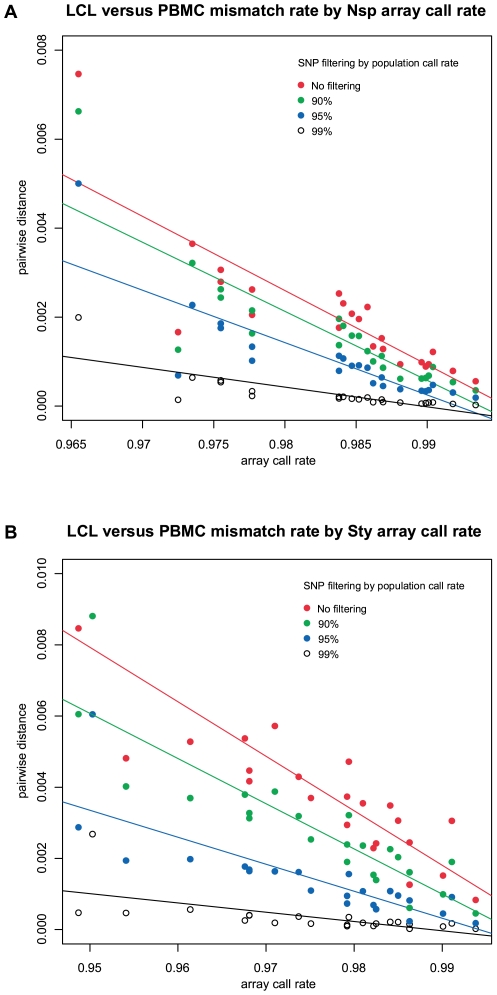
Effect of genotype array call rate on LCL versus PBMC genotypic fidelity. Relationship between genotypic fidelity of LCL and PBMC pairs (mismatch rate, estimated using pairwise distance) and the array call rate. For each LCL versus PBMC genotypic pair, we compared the LCL versus PBMC mismatch rate to the lesser of the two array call rates. Mismatch rates are estimated at different levels of SNP filtering by population call rate: no filtering, 90%, 95%, and 99% percent population call rate.

Genotypic fidelity (SNP genotype concordance) between LCL and PBMC varied across sample pairs, with mismatch rates ranging from 0.175% to 0.664% (with no filtering). To investigate the source of this variation, we compared the array call rate to the mismatch rate. For each LCL versus PBMC pair, we compared the LCL versus PBMC mismatch rate to the lesser of the two array call rates (we assumed the lesser array call rate was more likely to be the source of potential genotypic mismatch). There was a strong relationship between array call rate and mismatch rate, and the relationship is seen for both Nsp and Sty arrays (Figure 3). The correlation was strongest with no filtering by population call rate (Table 3; Nsp array: slope = −0.167, r^2^ = 0.70, *P* = 6.42×10^−7^), and is alleviated with increasingly stringent filtering by population call rate, as seen at the 99% population call rate (Table 3; Nsp array: slope = −0.044, r^2^ = 0.60, *P* = 1.43×10^−5^).

**Table 3:** Relationship between array call rate and LCL versus PBMC mismatch rate, for Nsp and Sty arrays, at different levels of SNP filtering by population call rate.

LCL versus PBMC mismatch rate by Array call rate			
Nsp 250 k SNP array			
population call rate filtering level	slope	r2	*P*
No filtering	−0.1668	0.7	6.42×10−7
≥90%	−0.1552	0.71	4.05×10−7
≥95%	−0.1177	0.69	1.06×10−6
≥99%	−0.0443	0.6	1.43×10−5
Sty 250 k SNP array			
population call rate filtering level	slope	r2	*P*
No filtering	−0.1531	0.77	4.41×10−8
≥90%	−0.127	0.78	1.92×10−8
≥95%	−0.0758	0.65	3.35×10−6
≥99%	−0.0262	0.39	1.38×10−3

### Departures from Hardy-Weinberg equilibrium

We compared the numbers of SNPs that depart from HWE in PBMC and LCL sample populations after filtering by population call rate and removing replicate samples. While a trend existed toward increased SNPs deviating from HWE in LCL samples, the differences were not statistically significant (*e.g.*, comparing the number of SNPs removed from LCL and PBMC sample populations, with HWE filtering at *P*<0.05, from populations with no call rate filtering, showed Χ^2^ = 0.12, *P* = 0.73), and more stringent population call rate filtering alleviated the discrepancy (Table 4).

**Table 4:** Filtering SNPs from PBMC and LCL (paired) samples based on departure from Hardy-Weinberg equilibrium at three levels of statistical significance, *P*<0.05, 0.01, and 0.001.

		PBMC 16	LCL 16	PBMC 16	LCL 16	Difference*	Chi-squared *P*
	starting SNPs	SNPs remaining		SNPs removed			
No filtering	500568						
HWE *P*	<0.05	484630	484568	15938	**16000**	−62	0.73
	<0.01	493254	493239	7314	**7329**	−15	0.9
	<0.001	495372	495387	**5196**	5181	15	0.88
≥90%	491525						
HWE *P*	<0.05	475867	475804	15658	**15721**	−63	0.72
	<0.01	484301	484293	7224	**7232**	−8	0.95
	<0.001	486350	486370	**5175**	5155	20	0.84
≥95%	458913						
HWE *P*	<0.05	444109	444075	14804	**14838**	−34	0.84
	<0.01	451912	451926	**7001**	6987	14	0.91
	<0.001	453829	453845	**5084**	5068	16	0.87
≥99%	311241						
HWE *P*	<0.05	299923	299915	11318	**11326**	−8	0.96
	<0.01	305355	305358	**5886**	5883	3	0.98
	<0.001	306808	306814	**4433**	4427	6	0.95

Bold text indicates the greater number of SNPs removed by Hardy-Weinberg filtering, in comparisons of PBMC and LCL sample populations.

* Difference between PBMC and LCL 16 in the number of SNPs removed by Hardy-Weinberg equilibrium filtering.

## Discussion

Here we report a study of SNP genotype fidelity between PBMC and EBV-transformed lymphoblastoid cell lines. We genotyped 16 PBMC and LCL pairs with the Affymetrix 500 k array (with three replicates genotyped, for 19 total comparisons). We estimated mean genotypic mismatch rates (estimated with pairwise genetic distances) and compared these rates to genotyping error rates reported by Affymetrix and also to mismatch rates seen in control pairs of duplicate genotyping of identical DNA samples. We also evaluated the effect of various levels of SNP quality control based on population call rate, and estimated mismatch rates seen with no SNP filtering, and with filtering those SNPs not called in 90%, 95%, and 99% or greater of individuals in a larger (N = 210) population. We found PBMC and LCL mismatch rates to be within ranges reported by Affymetrix for duplicate genotyping, when filtered at 95% or greater population call rate. PBMC and LCL mismatch rates are also not significantly greater than rates seen in control pairs.

We next compared mismatch rates seen for the Nsp and Sty 250 k subarrays, and found that rates for individual pairs were not correlated across subarrays, suggesting that the genotypic discrepancies observed are most likely due to technical artifacts of the genotyping process rather than the EBV transformation process. If the LCL genomic DNA contained gentoypic errors, mismatch rates would be consistent in Nsp and Sty arrays across the individuals examined. Additionally, the genotypic mismatch rate is highly correlated with the array call rate, and this relationship is seen for both Nsp and Sty arrays.

Genomic deletions in LCL have been hypothesized to result in an increased number of SNPs that depart from Hardy-Weinberg equilibrium, owing to a decreased number of heterozygotes. We tested for increased numbers of SNPs that depart from HWE in comparisons of PBMC and LCL samples, and while a trend existed in this direction, it was not statistically supported. We conclude that LCL are likely to have no apparent gross genotypic errors due to the process of EBV-transformation, and that SNP genotypes assayed from LCL may, with stringent quality control, be considered robust.
